# Do Drains Make a Difference? Assessing Ecchymosis and Edema After Osteotomies in Rhinoplasty

**DOI:** 10.1093/asjof/ojag138.011

**Published:** 2026-07-24

**Authors:** Sumun Khetpal, Jose Segura-Bermudez, Ava Jafanpour, Yasmine Ibrahim, Harsh Patel, Justin Dang, Jason Roostaeian

## Abstract

**Purpose/Background:**

Rhinoplasty is a complex operation that requires careful attention towards not only executing surgical maneuvers, but also analyzing their collective impact for optimal aesthetic and functional results. This relies on excellent surgical visibility, which entails minimal bleeding and clear dissection planes. While strategies such as permissive hypotension, perioperative steroids, and tranexamic acid (TXA) administration have been widely adopted to reduce intraoperative bleeding and postoperative morbidity, osteotomies remain a major source of mean arterial pressure (MAP) fluctuations and soft-tissue trauma. Moreover, disruption of the angular artery and its branches, fracture propagation, and manipulation of the osteocartilaginous framework can trigger cytokine release and increase capillary permeability, collectively contributing to postoperative edema and ecchymosis. To mitigate these effects, the senior author incorporated the use of drains placed immediately after osteotomies as an adjunct technique to reduce edema and ecchymosis and improve early recovery.

**Methods:**

A retrospective cohort study was conducted among patients undergoing primary rhinoplasty with osteotomies by the senior author. Patients who had a history of anticoagulant use, prior rhinoplasty, hypertension, and chronic systemic diseases were excluded. Patients were sorted into three respective cohorts: a) patients who did not have drains following osteotomy (Cohort 1), b) those who received drains upon conclusion of the rhinoplasty, (Cohort 2), and c) those who received drains upon completion of osteotomies (Cohort 3).

Demographic variables (age, gender) and TXA use (local, intravenous) were recorded. All patients underwent standardized postoperative photography. Frontal one-week postoperative photographs were randomized and assessed by three independent raters blinded to cohort assignment. Edema and ecchymosis were graded using validated 0–4 scales. Edema scoring was based on eyelid opening from fully open to fully closed, while ecchymosis scoring was based on the horizontal extent of bruising from the medial canthus to the lateral orbital rim.

**Results:**

A total of 45 patients were included. The average patient age was 30 years old, and mean followup at the time of follow-up was 7 days. There were no significant differences in demographic variables or TXA use between cohorts. Ecchymosis scores differed significantly across the three groups. Post-hoc pairwise analysis demonstrated that Cohort 3, in which drains were placed immediately following osteotomy, exhibited significantly lower mean ecchymosis scores compared with Cohort 1 (p = 0.0093) and Cohort 2 (p = 0.0315). In contrast, there was no significant difference between Cohort 1 and Cohort 2 (p = 0.887), suggesting that delayed drain placement does not meaningfully alter postoperative bruising. Edema scores were not significantly different between cohorts (p = 0.463), indicating that drain placement specifically influences ecchymosis rather than generalized swelling.

**Conclusion:**

This study demonstrates that the timing of intra-operative drain placement is critical: drains placed immediately after osteotomies significantly reduce postoperative ecchymosis. These findings highlight a simple, low-risk maneuver that may improve early post-operative appearance and potentially enhance surgical visualization and patient satisfaction.

